# In Situ Thermoset Cure Sensing: A Review of Correlation Methods

**DOI:** 10.3390/polym14152978

**Published:** 2022-07-22

**Authors:** Molly Hall, Xuesen Zeng, Tristan Shelley, Peter Schubel

**Affiliations:** Centre for Future Materials, University of Southern Queensland, Toowoomba, QLD 4350, Australia; molly.hall@usq.edu.au (M.H.); tristan.shelley@usq.edu.au (T.S.); peter.schubel@usq.edu.au (P.S.)

**Keywords:** composite manufacturing, thermosetting polymers, cure behaviour, process monitoring, in situ cure monitoring, sensors

## Abstract

Thermoset polymer composites have increased in use across multiple industries, with recent applications consisting of high-complexity and large-scale parts. As applications expand, the emphasis on accurate process-monitoring techniques has increased, with a variety of in situ cure-monitoring sensors being investigated by various research teams. To date, a wide range of data analysis techniques have been used to correlate data collected from thermocouple, dielectric, ultrasonic, and fibre-optic sensors to information on the material cure state. The methods used in existing publications have not been explicitly differentiated between, nor have they been directly compared. This paper provides a critical review of the different data collection and cure state correlation methods for these sensor types. The review includes details of the relevant sensor configurations and governing equations, material combinations, data verification techniques, identified potential research gaps, and areas of improvement. A wide range of both qualitative and quantitative analysis methods are discussed for each sensing technology. Critical analysis is provided on the capability and limitations of these methods to directly identify cure state information for the materials under investigation. This paper aims to provide the reader with sufficient background on available analysis techniques to assist in selecting the most appropriate method for the application.

## 1. Introduction

Advanced thermoset polymer composites are implemented in a variety of industries, such as in civil [1,2] and energy [3,4,5] and in recreational and naval marine applications [6,7,8,9,10] as well as in performance automobiles [11,12] and in aerospace applications [13,14,15]. The adoption of thermoset materials has increased in recent years due to the tailorability of part properties and wide variety of manufacturing techniques and achievable geometries. Thermosets can be formed as unreinforced plastics or reinforced composites via injection and compression moulding [16] and resin infusion [17] or using automated laydown techniques [18]. The parts must then go through a cure cycle, commonly under elevated temperature and/or pressure conditions, such as in an as autoclave or oven [19,20]. Recently, research on fibre-reinforced polymer (FRP) composites has trended towards the development of high-quality parts that are up to tens of metres long [21] and more than 2 cm thick [22], with emphasis on optimising the processing conditions when making these complex parts [23].

Composite parts are susceptible to a variety of quality issues, such as fibre displacements, voids and porosity, geometric deformations, and inconsistent chemical reactions or polymerisations [24]. These final-part variations are frequently a result of manufacturing uncertainty stemming from either variation in the raw materials or in the processing conditions and environment [25]. In advanced composite applications, it is a critical quality objective to achieve a specified resin cure state, as the completion of the polymer conversion process is directly linked to the mechanical performance of the final product [26]. To capture the effects of this variability, it becomes necessary to monitor the cure process for each individual part.

The final cure state of a thermoset part is typically evaluated using either a quantifiable degree of cure, specified as a percentage of the chemical reaction that has been completed, or by reaching a threshold value for the glass transition temperature (*T_g_*) [27,28]. The degree of reaction or polymerisation can be analysed off-line, where testing is conducted externally to the manufacturing process, or in-line, where a sensor is integrated directly into the manufacturing process and captures live data [29]. The advantages of the in-line or in situ monitoring of composite processing are the ability to monitor the process in real time [30] and the potential to actively control the process as it occurs [31,32]. Further, some major limitations of off-line cure evaluation are that it may require destructive testing, cannot perfectly replicate the process conditions during part of the cure, and cannot be used to update the process conditions in real time.

**Figure 1:**
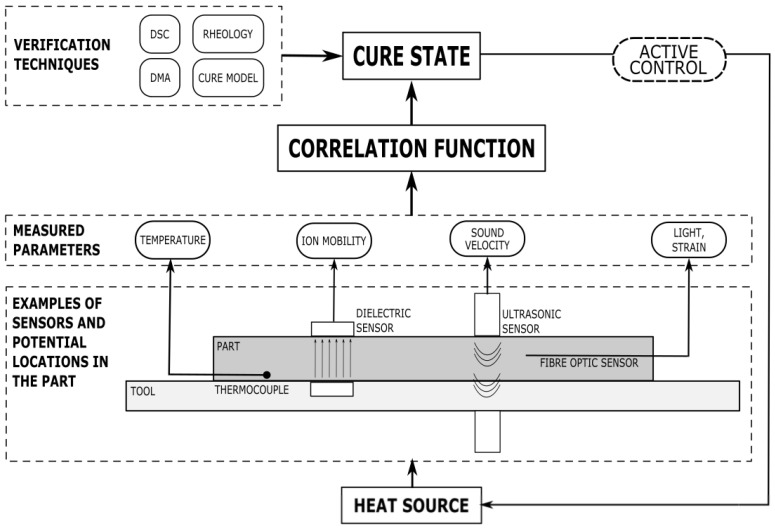
An overview of types of cure sensors, their placement, measured parameters, and verification techniques. The data flow process for an active control system is also proposed, with the sensors being correlated to cure state information during live processing, which can subsequently be used to actively alter the processing conditions.

This paper will briefly review the established off-line cure-monitoring techniques such as Dynamic Scanning Calorimetry (DSC), Dynamic Mechanical Analysis (DMA), and Dynamic Rheometry. A deeper evaluation of direct sensing technologies for in-line curing is then presented, specifically of thermocouple, dielectric, ultrasonic, and fibre-optic sensors. Extensive reviews have been completed regarding the capabilities and limitations of these sensors for composite process and cure monitoring [33,34]; however, a critical review of the correlation methods of these techniques has not been carried out to date. Each type of sensor monitors different parameters, and data analysis must be conducted to convert these parameters into information pertaining to the material cure state, with an example of the data flow and analysis procedure being shown in Figure 1. In this paper, a critical review of correlation processes for four in-line sensing technologies is presented. Special focus has been placed on the specific sensor type and material configuration, the results of the correlation analysis, and how the analysis has been verified for accuracy. The technologies are then evaluated for how effectively they monitor composite cure processes and how appropriate they may be for high-performance applications.

## 2. Off-Line Cure Analysis

Off-line cure analysis techniques are frequently used to characterise new material systems or as a quality control evaluation of an existing cured part. Material characterisation enables researchers to build a model of the material that can then be used in process simulations. For example, a research team characterised Hexcel RTM6 using DSC [35] and rheometric [36] analysis to develop a kinetic and a chemosviscosity model of the tested epoxy. Three common off-line analysis techniques are discussed here, including their governing equations and identification principles for cure state information. Other analysis techniques, such as Fourier Transform Infrared (FTIR) [37] and Raman spectroscopy [38], are used for polymer analysis; these will not be discussed further.

### 2.1. Dynamic Scanning Calorimetry (DSC)

DSC measures heat flow in a sample when it is subjected to isothermal or non-isothermal temperature conditions. By integrating the peak of the heat flow (*H*) versus the time curve and dividing it by the total heat of reaction (*H_R_*), we can calculate the degree of cure (*α*), as shown in Equation (1).

(1) $$\alpha =\frac{\int_{0}^{t}Hdt}{H_{R}}$$

There is an extensive amount of literature on the use of DSC to characterise cure reactions [39,40,41,42], and the procedure for kinetic parameter determination is detailed in standards such as ASTM E 2070, which contains methods for kinetic parameters by differential scanning calorimetry using isothermal methods [43]. DSC can also be used to measure thermoset cure reactions [44] and to calculate the degree of cure of an existing cured part. The residual heat of reaction can be measured for a cured sample, which allows for the calculation of the actual degree of cure of the part based on a known total heat of reaction for the material. DSC analysis is used to validate the results of new sensing technologies and will be mentioned throughout this paper as one of the main verification techniques.

### 2.2. Dynamic Mechanical Analysis (DMA)

**Figure 2:**
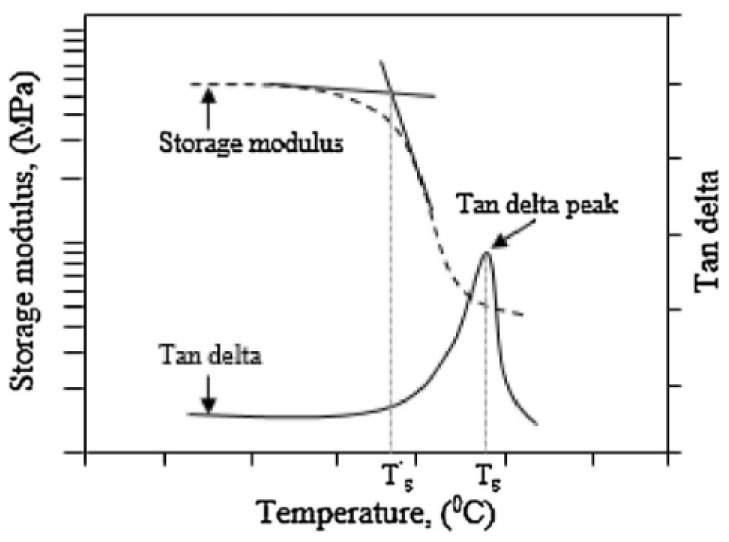
Graphic of the calculation of *T_g_* using an *E′* modulus curve (**left-hand axis**) and *tanδ* curve (**right-hand axis**) from a DMA test. Reprinted with permission from Ref. [46]. 2016, Elsevier.

The DMA of composite parts utilises a dual cantilever beam configuration in which a sample is oscillated at a set frequency through a set temperature range. The elastic modulus is evaluated throughout the test; specifically, the storage modulus (*E′*) component, the loss modulus component (*E″*), and the *tanδ*, which is calculated as the ratio of the loss to the storage moduli, are considered. The main output of a DMA test is the *T_g_*, which is calculated as the midpoint of the drop in the storage modulus. ASTM D 7028, which provides methods for *T_g_* determination in Polymer Matrix Composites via DMA [45] details the process for the calculation of *T_g_* by identifying the intersection of the tangent lines around the drop in *E′*, as shown in Figure 2.

DMA has been used to identify the cure state of many materials, such as phenolics [47] and epoxies [48]. Such as with DSC, DMA testing is used throughout this paper to verify the *T_g_* calculations of the in-line sensing techniques.

### 2.3. Dynamic Rheometry

The dynamic rheometry of thermoset composites typically occurs in a parallel-plate oscillating configuration, with the purposes of monitoring the change in the shear modulus under a set temperature range. Like DMA, rheometric testing evaluates the shear storage modulus (*G′*), the loss modulus (*G″*), and *tanδ*, which is once again the ratio of loss to the storage moduli. From these values, the complex viscosity (η*) can be calculated by Equation (2) using the complex modulus (G*) and oscillating frequency (*ω*):

(2) $$\eta^{*}=\frac{G^{*}}{\omega }$$

While this does not specifically relate to the final cure state of a thermoset polymer, resin viscosity can be a critical parameter during processing.

**Figure 3:**
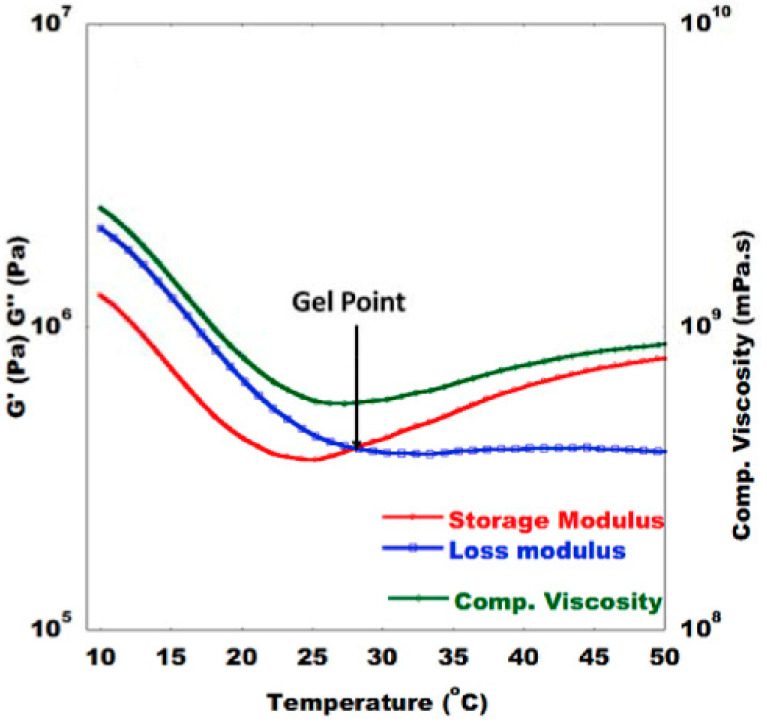
Example of how gel time can be determined by identifying where *G′* and *G″* cross. Reprinted with permission from Ref. [50]. 2019, Elsevier.

Regarding the cure state, the gel point can be defined in multiple ways in accordance with the rules of ASTM D 7750, which contains methods for evaluating cure behaviour of thermosetting resins [49], an example of which is displayed in Figure 3. Depending on the interactions of fibre and resin, the gel point can be defined as the intersection of *G′* and *G″*, the peak of *G″*, the peak of *tanδ*, a sudden rapid increase in *G′*, or a sudden drop in *tanδ*.

The main challenge of rheometric cure monitoring is that during the crosslinking and solidification process, the viscosity trends towards infinite, so later step cure stages cannot be monitored. Despite this, rheometry has been used to evaluate viscosity and cure progression for several thermoset polymers [42,51] and is also used as a verification technique for the sensors discussed in this paper.

## 3. In-Line Cure Monitoring Sensor Correlations

### 3.1. Thermocouple Sensors

#### 3.1.1. Sensor Background and Governing Equations

There are a variety of sensors that are capable of monitoring the thermal properties of composite cure processing, including thermocouples (TCs), infrared thermographers (IRT) [52], heat flux sensors [22], and resistance temperature detectors (RTDs) [53]. While this paper specifically focuses on thermocouples, alternative temperature sensors have been reviewed [29], including details on their functionality, capabilities, and limitations.

**Figure 4:**
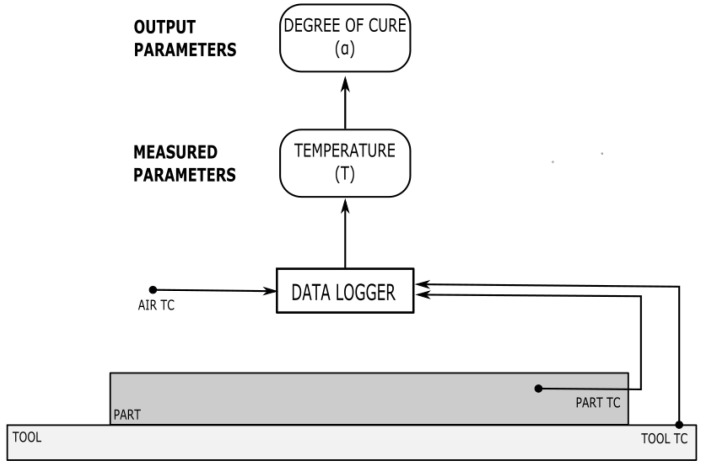
An overview of thermocouple placements and the data collection process flow.

Temperature is one of the most common parameters to measure during composite processing, as the time–temperature–transformation relationship of thermosetting polymers is well established [54], and most thermoset resins are cured under the application of a specific heating cycle [55]. Temperature monitoring of both the environmental conditions, for example, the oven or autoclave air temperature, and the material of choice is extremely important. Most composite processes include an air TC to account for environmental uncertainty, such as the natural fluctuations in the equipment over time. Additionally, the actual temperature experienced by the part is critical for cure monitoring, as many thermoset polymers tend to experience exothermic events, or a temperature increases due to the release of heat energy during the chemical reaction. Material uncertainties such as slight variations in the raw material; the initial degree of cure; and the material age, storage conditions, and resin content can all impact the likelihood and peak temperature of an exotherm [25]. For this reason, simply monitoring the equipment temperature may not be sufficient to identify and predict the exact temperature profile that the part is experiencing. Thermocouples are commonly placed in one or more representative locations: in the part, on or in the tool, and in the air, to monitor the environmental conditions. These locations and an overview of the parameter’s monitoring process is shown in Figure 4.

Thermocouples comprise two different metal wires with known Seebeck voltages, which are welded or twisted into a junction at one end and separated at the other. The monitoring temperature (*T_m_*) at the welded junction can be calculated using Equation (3) using the Seebeck coefficient (*S*), measured voltage (*V*), and reference temperature at the open junction (*T_r_*).

(3) $$T_{m}=\frac{V}{S}+T_{r}$$

This equation is used to reliably calculate the temperature being experienced by the material in question. Using this temperature profile, the material properties can then be predicted according to the methods detailed in the following section.

#### 3.1.2. Correlation Functions

An overwhelming amount of literature exists on the use of thermocouples and temperature devices to monitor the progression of thermoset cure. For example, TCs and IRTs have been used to monitor temperature distributions and exotherms of carbon fibre–epoxy composites and other polymers [56,57] and to monitor part cure as a method to validate simulation results [22]. Thermocouples are used to monitor processing and part temperatures in most composite cure studies, including in almost every paper mentioned in this review, due to their fundamental nature.

The most reliable method of directly correlating the measured temperature to the material degree of cure (*α*) is by evaluating the thermo-kinetic model of the material, which roughly follows the formula in Equation (4):

(4) $$\frac{d\alpha }{dt}=K\left(T\right)f\left(\alpha \right)$$

in which $\frac{d\alpha }{dt}$ indicates the change in the degree of cure with respect to time, the component *K*(*T*) represents the temperature dependency component, and *f*(*α*) represents the reaction model component. *K*(*T*) follows an Arrhenius dependence and can be calculated using Equation (5) using the pre-exponential factor (*A*), activation energy (*E*), universal gas constant (*R*), and the temperature:

(5) $$K\left(T\right)=Ae^{\frac{-E}{RT}}$$

The reaction model component, *f*(*α*), is specific to the material in question. Many reaction models have been proposed, with a comprehensive overview published by Yousefi et al. [44]. Examples include a simple nth-order rate equation [58], the autocatalytic model [59], or model-free kinetic analysis [60]. While some models can be broadly applied to material classes such as epoxies or polyesters, it is also best to conduct a kinetic analysis of each specific material component to increase the accuracy of the results.

**Figure 5:**
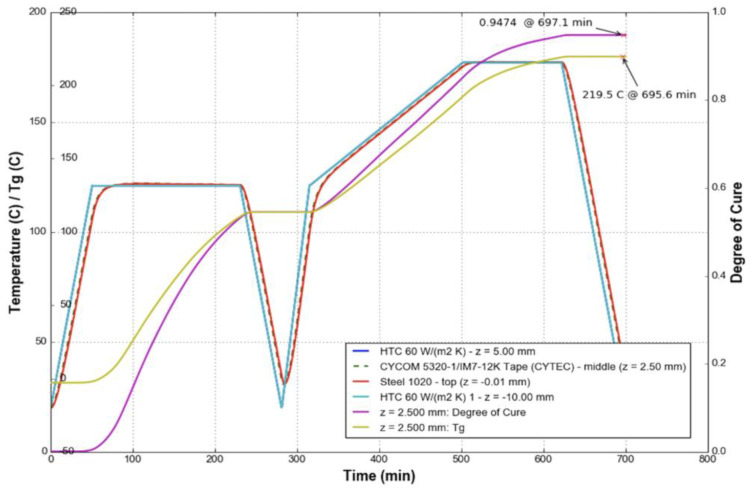
An example of the manufacturer’s recommended cure cycle for Cycom^®^ 5320-1 Prepreg and it’s resultant predicted final properties (degree of cure and *T_g_*).

In practice, the temperature profile of a composite part can be verified against the kinetic model or against a simulation that incorporates the kinetic model [61]. Once the temperature profile is verified to produce an acceptable degree of cure, it is typical to simply verify that the temperature parameters are met for each process cycle. For applications that may not have the capacity, need, or interest in completing such a process verification, it is common to follow the manufacturers’ recommended cure cycle as found in the technical data sheet for most commercial thermosets or for composite materials, such as for Solvay Cycom^®^ 5320-1 Prepreg [62]. The material manufacturer typically specifies one or more recommended cure cycles that will ensure that the part reaches a fully cured state. In this case, a temperature reading is taken from a representative location that is either in or on the part, on the tool, or elsewhere in the oven. The main verification method for quality control is to check the temperature as a function of time compared to the recommended cure cycle requirements, as shown in Figure 5, rather than to calculate a specific degree of cure for each individual part. This quality control step ensures that the cured material meets the minimum threshold for mechanical performance, as the required engineering properties can only be met in fully cured parts [63]. It should be noted that the definition of “fully cured” varies based on the specific material and application.

#### 3.1.3. Summary and Future Work

Thermocouples are the most common and widely used sensing technology for composite curing and process monitoring. They monitor not only the cure state, which is measured as the degree of cure, but are also able to monitor other critical process events such as temperature overshoots caused by exothermic events. Additionally, they are frequently required to be used in coordination with other sensing devices, such as those detailed in the below sections, to normalise for temperature effects [64,65] or as a supplemental monitoring technique for data collection. Thermocouples have also been used to monitor resin filling for infusion processes [66] and are commonly used to monitor temperature applications during process optimisation activities [23,67,68]. A major challenge of thermocouples is that to directly measure the material state, they must be embedded into the part, and some applications (such as those which require specific surface finishes) are unable to accept embedded sensors.

### 3.2. Dielectric Sensors

#### 3.2.1. Sensor Background and Governing Equations

In recent decades, dielectric sensors have been investigated as a new method of in situ cure monitoring for thermoset composite materials due to their versatility and range of available configurations, both when purchased off the shelf and when custom-designed. The three most common types of dielectric sensors are parallel-plate, interdigital, and tool-mounted. Each of these has benefits and limitations, which have been discussed in depth elsewhere [33,34]. For example, parallel-plate dielectrics can detect through-thickness measurements that would otherwise require interdigital sensors to be embedded throughout the thickness of a part. Interdigital and tool-mounted sensors only take measurements of the surface that they are directly in contact with; however, interdigital sensors are commonly used invasively, making them less optimal for industries with stringent quality requirements.

Dielectric sensors work on the principle of monitoring dipole and ion movement within a material under a time-varying electric field (*E*). The alignment and relaxation of the charged particles within the sample are monitored by the sensor in the form of a capacitive (*C*) and resistive (*R*) response [69]. These values are used to calculate the dielectric parameters to be referenced throughout this paper. Thermoset curing can be evaluated using these parameters due to the time-, temperature-, and frequency-dependent response of the dielectric sensor. The dielectric sensor captures the change in ion mobility, which directly relates to the cure state of the material as it crosslinks. It should be noted that some of the variable representations in this paper may differ from the cited sources to maintain the consistency of the variable meanings used in the following governing equations and correlations.

Permittivity (*ε′*) is calculated in Equation (6) using capacitance, electrode spacing (*L*), the electrode area (*A*), and the permittivity of free space (*ε*_0_ = 8.854 × 10^−12^ F/m), as derived from [70]:

(6) $$\;\varepsilon^{\prime }=\frac{CL}{\varepsilon_{0}A}$$

Dielectric loss (*ε″*) is calculated in Equation (7) using resistance, the electrical excitation frequency (ω), electrode spacing and area, and the permittivity of free space, as derived from [70]:

(7) $$\varepsilon^{\prime \prime }=\frac{L}{R\omega A\varepsilon_{0}}$$

Impedance (*Z*) is calculated in Equation (8) with the resistance, excitation frequency, and conductance, with *j* as the imaginary component [71]:

(8) $$Z=\frac{1}{\frac{1}{R}+j\omega C}$$

Ion conductivity (*σ*), which is related to the inverse relationship of ion viscosity and frequency-independent resistivity (ρ), is calculated in Equation (9) using resistance, electrode spacing, and electrode area [71]:

(9) $$\rho =\frac{1}{\sigma }=\frac{RA}{L}$$

The dissipation factor (*D*), also known as *tanδ*, can be calculated in Equation (10) using the permittivity and dielectric loss or the resistance, capacitance, and excitation frequency [72]:

(10) $$D=\tan\delta =\frac{\varepsilon^{\prime \prime }}{\varepsilon^{\prime }}=\frac{1}{\omega RC}$$

**Figure 6:**
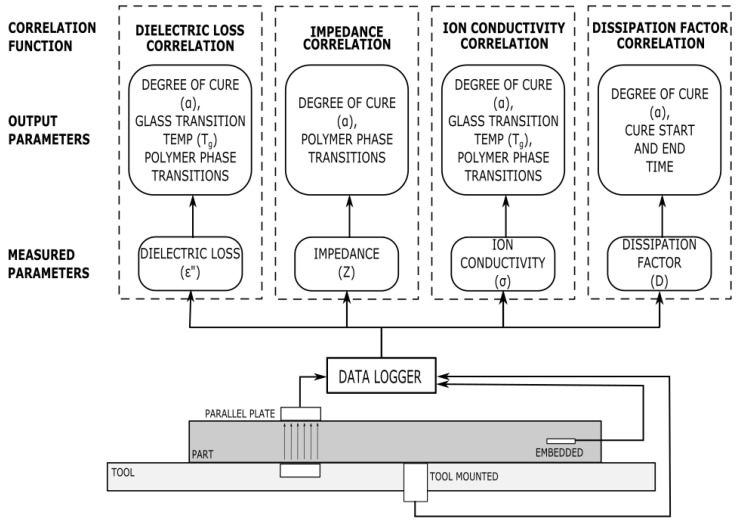
An overview of dielectric sensor correlation methods, including a visual depiction of the types of dielectric sensor, the parameters they measure, and how the parameters are converted into cure information.

While the dielectric response provides a great deal of information, it does not directly relate to information about the cure state of a thermoset polymer. A correlation function is needed to relate the dielectric properties to the state of the chemical reaction, specifically the degree of cure and glass transition temperature. The data may be interpreted qualitatively by evaluating artefacts from a graph or quantitatively by deriving equations. The data must also be corroborated using techniques that are currently known to provide insight into the cure state of a thermoset polymer: typically thermochemical or rheometric testing. Examples of these methods are provided in the following section, with an overview shown in Figure 6.

#### 3.2.2. Correlation Functions

There are many methods for correlating dielectric signals with the degree of chemical reaction that has occurred in the resin or composite. Common methods and their variants will be discussed in this section, including the correlation functions and the supplemental testing techniques.

##### Dielectric Loss Correlation

**Figure 7:**
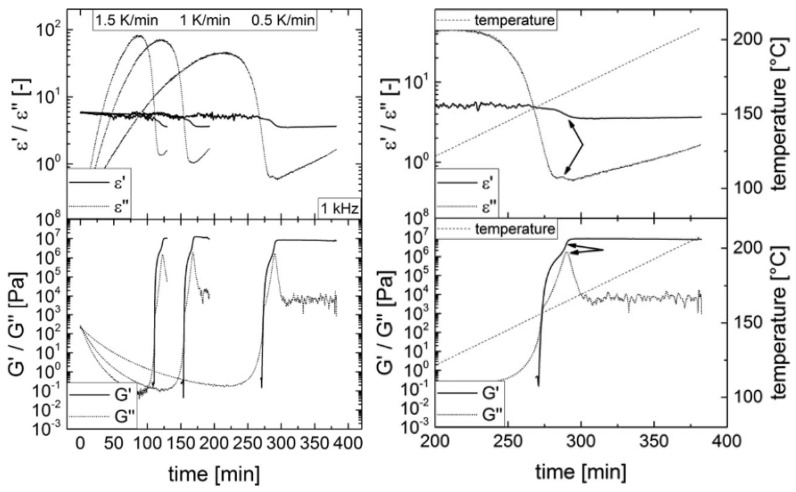
Comparison of dielectric loss to rheological storage and loss as a method to identify *T_g_* (as identified by arrows in the right-hand image). Reprinted with permission from Ref. [74] 2018, John Wiley and Sons.

Fournier et al. [73] used a dielectric loss correlation through their work evaluating neat epoxy resin using parallel-plate dielectric sensors. The dielectric loss factor (*ε″*), which can be calculated from Equation (7), was used to predict vitrification by identifying the time of maximum loss for each experimental frequency. Dielectric loss correlations have also been used to identify the gel point and have been verified through comparison to rheology data [74]. Using neat RTM6 epoxy monitored by a tool-mounted dielectric sensor, the glass transition temperature was determined as the local maximum of the dielectric loss graph. Additionally, the crossover point between the permittivity and dielectric loss can be demonstrated to indicate the gel point. This has been correlated to rheology test data and specifically to the crossover point of the storage modulus and the loss modulus, *G′* and *G″*, as seen in Figure 7.

From a quantitative perspective, Hardis et al. proposed an equation for the degree of cure based on the progression of dielectric loss during the cure of an epoxy monitored with parallel-plate dielectrics [75]. The equation for degree of cure (*α*) with respect to time is stated in Equation (11):

(11) $$\alpha \left(t\right)=\frac{\log\varepsilon_{0}^{\prime \prime }-\log\varepsilon_{t}^{\prime \prime }}{\log\varepsilon_{0}^{\prime \prime }-\log\varepsilon_{\infty }^{\prime \prime }}$$

where the subscripts *ε″* represent the dielectric loss at start of cure (*ε*_0_″), at time *t* (*ε_t_″*), and at cure completion (*ε_∞_″*). The degree of cure generated from this equation aligned well with degree of cure measurements determined from DSC and Raman spectroscopy.

##### Impedance Correlation

**Figure 8:**
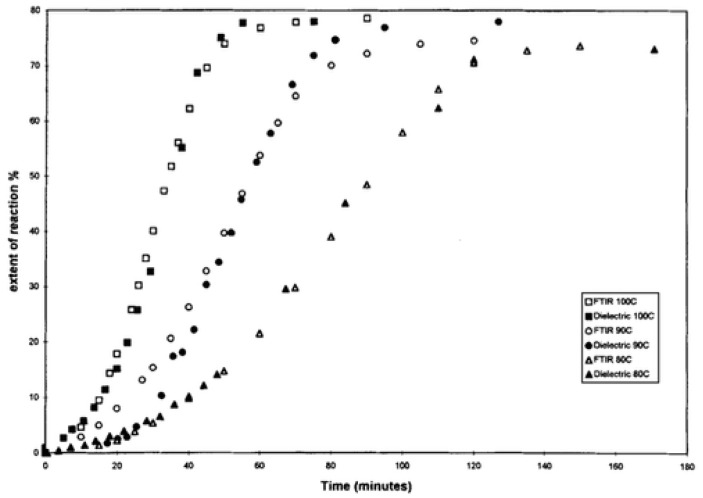
Comparison of degree of cure between dielectric and FTIR analysis represented as the extent of reaction (%) versus time (minutes), with curves indicated at various temperatures. Reprinted with permission from Ref. [76] 2003, John Wiley and Sons.

Mijovic et al. used an impedance correlation to calculate the resistivity of a sample based on the monitored impedance signal (*Z*) calculated in Equation (8). Impedance was used to calculate resistivity (ρ), and then boundary conditions were evaluated to derive Equation (12) for the degree of cure [71,76]:

(12) $$\frac{\alpha }{\alpha_{m}}=\frac{\log\rho -\log\rho_{o}}{\log\rho_{m}-\log\rho_{o}}$$

in which *α_m_* represents the maximum degree of cure, and $\rho_{0}$ and $\rho_{\infty }$ represent the initial and maximum values of resistivity. The cure progression of neat epoxies was evaluated using this function, and graphs of the degree of cure versus time were compared successfully to those produced by HPLC and FTIR analysis, as shown in Figure 8. Further, the vitrification point was identified at the onset of the second step on the graph showing imaginary impedance (*Z″*) versus time, and this point was successfully correlated to the storage modulus (*G″)* peak from the corresponding rheological data.

**Figure 9:**
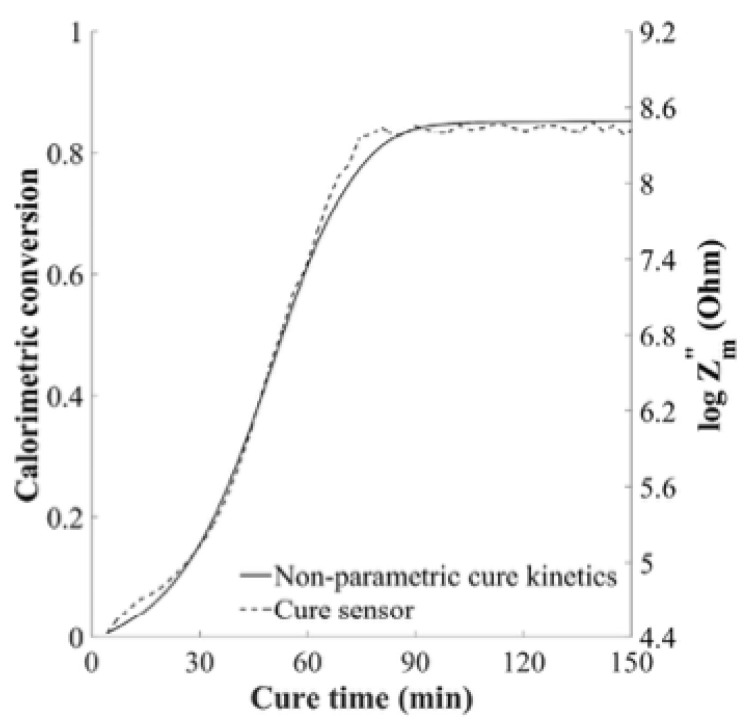
Comparison between *Z_m_″* and degree of cure generated from cure kinetics model [77].

This method has recently been used for determining the vitrification point of an RTM6 epoxy reinforced with carbon fibre [77,78]. In this method, the imaginary impedance (*Z″*), a component of Equation (8), is evaluated across multiple frequencies to eliminate the impact of the constant phase element, the second term of Equation (13):

(13) $$Z^{\prime \prime }=\frac{\omega CR^{2}}{1+\omega^{2}C^{2}R^{2}}+\frac{2}{{\left(A_{e}\omega \right)}^{n}}$$

in which *A_e_* and *n* are coefficients of the constant phase element. The first term of Equation (13) provides *Z_m_″*, or the material impedance, and a plot such as the one in Figure 9 has been overlaid on a graph of degree of cure derived from the material cure model. This qualitative comparison shows similar trends between the term *Z_m_″* and the degree of cure. Furthermore, the second step or shoulder region on the graph of *Z_m_″* versus time indicates the vitrification point. Studies by this research group were conducted using both a customized woven sensor for the cure monitoring and a lineal sensor for the flow monitoring of the resin infusion process. Interestingly, the lineal sensor configuration was also able to produce a cure signal that was reasonably similar to that produced by the cure sensor [78].

Similarly, evaluating the frequency spectra of the imaginary impedance has been used to draw a direct correlation to the degree of cure [79]. By applying linear regression to the graph of degree of cure versus log(*Z″_max_*) at temperature *T*, the *c* coefficients in Equation (14) can be determined:

(14) $$\log Z_{max}^{\prime \prime }=\left(c_{11}+c_{12}T\right)\alpha +c_{2}$$

**Figure 10:**
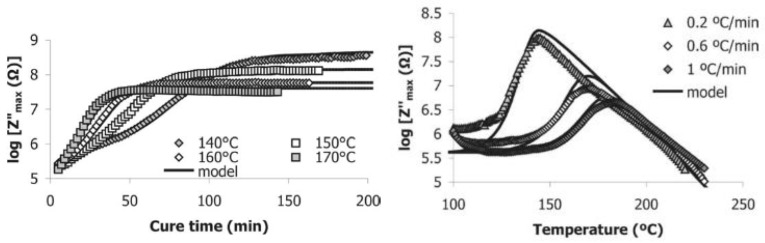
Comparison of experimental values of *Z″* with the proposed model for *Z″* for an isothermal cure (**left**) and a non-isothermal cure (**right**). Reprinted with permission from Ref. [79] 2005, Elsevier.

This equation was used to successfully model an isothermal cure cycle of RTM6 epoxy using an interdigitated dielectric sensor and a degree of cure prediction from the cure kinetics model. Figure 10 shows a comparison of this model to the experimental data of *Z″*. Furthermore, a non-isothermal cure was shown to fit the model quite closely, although with slightly more errors in the progression of the model.

##### Ion Conductivity Correlation

Ion conductivity correlations have been used the most frequently due to the connection of ion conductivity, and therefore ion viscosity, to the bulk polymer viscosity. In this section, various approaches are used based on whether the ion conductivity or ion viscosity, which is also known as the polymer resistivity (ρ), are being monitored.

**Figure 11:**
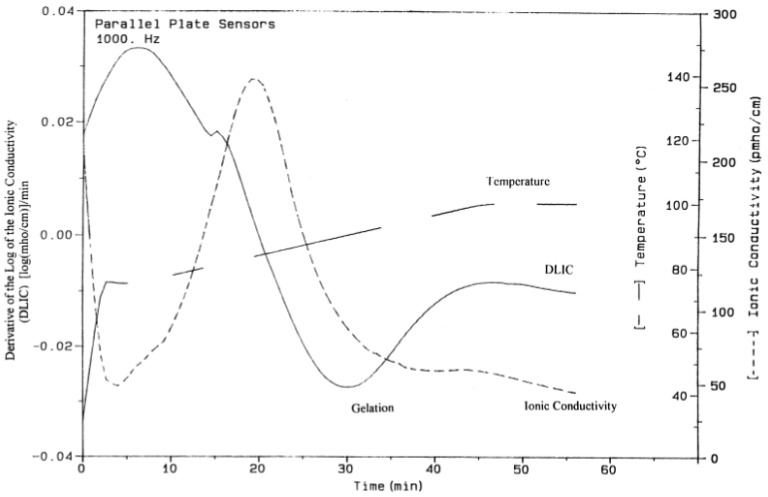
Gel point indicated on a DLIC curve. Reprinted with permission from Ref. [53] 2000, Elsevier.

Starting with ion conductivity, McIlhagger et al. determined the *T_g_* of an epoxy matrix reinforced by both glass and carbon fibres using signals generated from parallel-plate dielectric sensors [53,80]. The derivative of the log of the ionic conductivity, known as the DLIC, approaches zero as the sample approaches full cure. This cure point has been compared to DMA and DSC results in addition to being verified by tension and flexure mechanical performance tests to identify the peak of material performance, which occurs at full cure [80]. Additional critical points have been determined using a plot of the ionic conductivity. The maximum conductivity occurs at the point of minimum resin viscosity, which can be a critical point for out-of-autoclave and resin infusion processing, and as seen in Figure 11, the minimum point of DLIC indicates the onset of gelation [53]. McIlhagger et al. determined the minimum viscosity, gel point, and point of full cure with the data corroborated using DMA and DSC testing [53].

This correlation method has also been employed elsewhere, specifically in assigning the maximum value of ionic conductivity to the point of minimum polymer viscosity, the inflection point of the LIC after the peak viscosity relating to the onset of gel, and the maximum of dielectric loss corresponding to the onset of vitrification [56,81,82].

Yang et al. proposed Equation (15) as a method to calculate the *T_g_* of an epoxy resin using a miniature interdigital sensor to monitor ionic conductivity:

(15) $$T_{g}=\frac{\log G_{0}\left(T\right)-\log G\left(t\right)}{\log G_{0}\left(T\right)-\log G_{\infty }\left(T\right)}T_{g\infty }\left(T\right)$$

where *G*_0_*(T)* and *G_∞_(T)* are the temperature-dependent initial and final conductance, *G(t)* is the time-dependent conductance, and *T_g∞_(T)* is the *T_g_* calculation based on a cure kinetics model [83]. Through this in situ calculation of *T_g_* and use of the DiBenedetto equation, the degree of cure can be calculated as in Equation (16):

(16) $$\alpha =\frac{T_{g}-T_{g0}}{T_{g}-\lambda T_{g}-T_{g0}+\lambda T_{g\infty }}$$

which uses the *T_g_* values calculated from Equation (15) and *λ*, which is a ratio of the heat capacities as calculated during cure kinetics characterisation. This prediction has indicated a consistent trend, however an error of approximately 5–10% exists when compared with DSC.

Ion viscosity correlations are related to ion conductivity through the inverse relationship ρ=1/σ and is then correlated to polymer viscosity values through Equation (17):

(17) $$\rho =\frac{6\pi \eta r}{q^{2}n}$$

which uses polymer viscosity (*η*), ion particle size (*r*), ion charge (*q*), and ion concentration (*n*) [84]. As the ion viscosity thus has a direct relationship to polymer matrix viscosity, it is possible to understand key information regarding thermoset cure based on our knowledge of viscosity progression.

Boll et al. evaluated a carbon fibre/epoxy composite using a miniature embedded dielectric sensor by estimating that cure completion occurs when ρ reaches a plateau [84]. The cure state was then verified by completing a DSC evaluation of the cured part and by determining the degree of cure from the residual enthalpy. This method was also used by Moghaddam et al. when evaluating the effectiveness of their micro interdigitated sensor compared to current commercial sensors [85].

**Figure 12:**
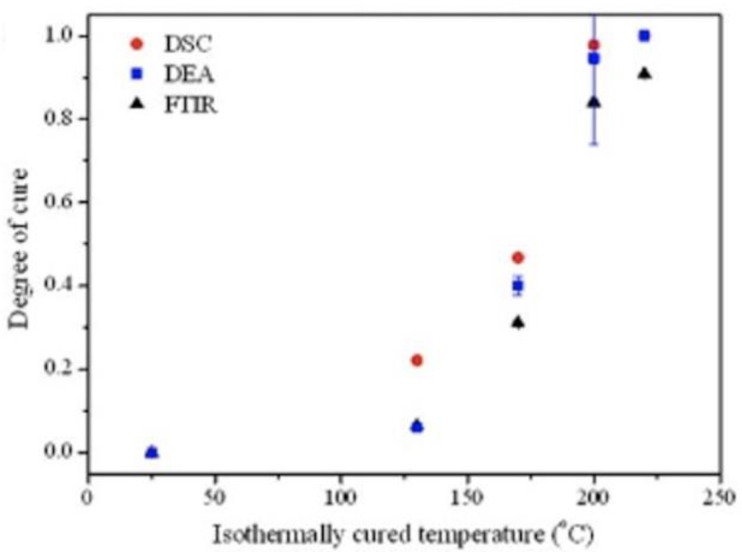
Comparison of degree of cure calculated from dielectric-monitored ion viscosity compared to DSC and FTIR analysis. Reprinted with permission from Ref. [86] 2017, John Wiley and Sons.

For a glass–epoxy prepreg monitored with a surface-mounted interdigitated electrode, Park established that the log of the ion viscosity had a linear relation to the cure temperature [86]. This enabled the calculation of Equation (18) for the degree of cure through a derivation of the DiBenedetto equation:

(18) $$\frac{\log\rho -\log\rho_{0}}{\log\rho_{\infty }-\log\rho_{0}}=\frac{T_{g}-T_{g0}}{T_{g\infty }-T_{g0}}=\frac{\lambda \alpha }{1-\left(1-\lambda \right)\alpha }$$

in which the subscript 0 indicates the initial condition, and ∞ indicates the fully cured condition. A comparison of the degree of cure calculated from Equation (18) to that derived from DSC and FTIR analysis is shown in Figure 12, with the DEA results being comparable to those of the other methods.

**Figure 13:**
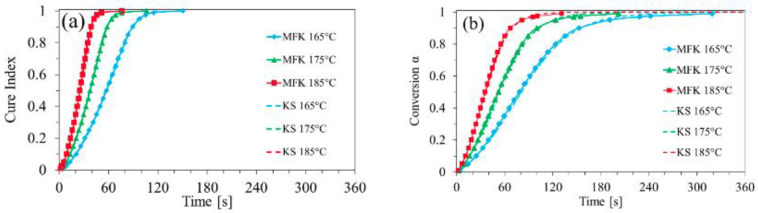
Comparison between cure index derived from DEA (**a**) and conversion derived from DSC (**b**) [87].

A similar equation for degree of cure was calculated from the ion viscosities in accordance with Equation (19):

(19) $$\alpha =\frac{\log\rho -\log\rho_{min}}{\log\rho_{max}-\log\rho_{min}}$$

in which the subscripts indicate the minimum and maximum ion viscosities measured during the cure. Franieck et al. evaluated Equation (19) for a silica-filled epoxy in which cure was monitored using a tool-mounted monotrode dielectric sensor [87]. The results from this analysis were compared to the degree of cure calculated from DSC, with limited success. While the DEA and DSC graphs follow similar trends, the DEA results are limited by the onset of vitrification, where the DSC results appear to better capture conversion during the diffusion-controlled period of cure. Figure 13 shows the differences in the results, with the DEA-calculated cure index operating on a shorter time scale than the DSC results.

Interestingly, Franieck et al. did not limit their investigation into dielectric cure monitoring and instead used the dielectric results to develop a kinetic model. The focus of this paper was to compare both the model-free and model-based kinetic equations derived from dielectric analysis with those derived from DSC results. In this they determined that the dielectric kinetic model aligned with the experimental data; however, as stated previously, the dielectric model and DSC model showed differences around the vitrification point.

##### Dissipation Factor Correlation

**Figure 14:**
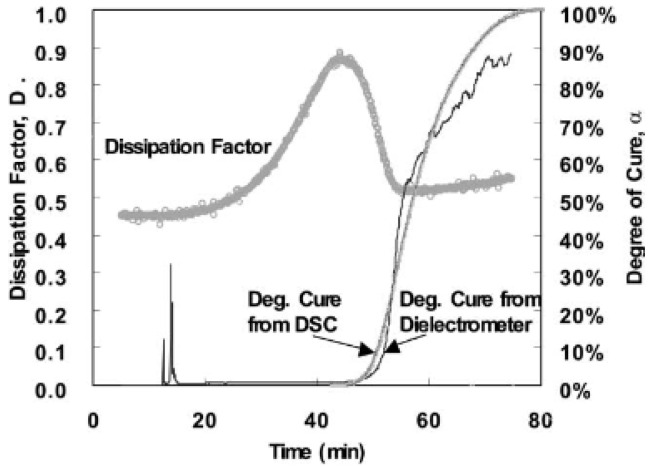
Comparison of degree of cure between dielectric testing and DSC. Reprinted with permission from Ref. [64] 2002, Elsevier.

Kim and Lee used a dissipation factor correlation, in which the dissipation factor was normalised for temperature effects, and an equation for the degree of cure was derived [64,88]. An interdigital dielectric sensor was used to monitor the resistance (*R*) and capacitance (*C*) of polyester–fibreglass and epoxy–fibreglass composites. The resistance and capacitance were used to calculate the dissipation factor following Equation (10). As the dissipation factor is a function of both the temperature and degree of cure of the matrix, the elimination of the temperature component will allow for the degree of cure to be calculated. The degree of cure determined from *D* was compared to that of DSC and demonstrated fair accuracy up to a cure level of approximately 70%, as seen in Figure 14.

Using the same method of eliminating the temperature effects, Equation (20) was derived to determine the degree of cure:

(20) $$\alpha =-\frac{1}{s}\log\left[\left(\frac{\log D-\log D_{o}}{q\left(T-T_{o}\right)}\right)-\frac{p}{q}\right]$$

in which the material parameters *D_o_*, *T_o_*, *p*, *q*, and *s* can be determined experimentally by following the procedure stated in [88].

Another method for evaluating the dissipation factor was calculated from an interdigital dielectric sensor reading and then used to determine the start and end points of cure for a carbon–epoxy composite [89]. The derivative of the dissipation factor was taken with respect to time, enabling the cure start time to be identified as the maximum of *dD/dt*, and the cure end time to be identified as *dD/dt* = 0.

#### 3.2.3. Summary and Future Work

Dielectric analysis shows much promise for the in-line cure monitoring of thermoset composites. There are many methods of correlating dielectric data to material transitions, such as the gel and vitrification points, and physical properties, such as *T_g_* and the degree of cure. Currently, a major gap in our understanding of dielectric cure analysis is which of these methods is the most accurate, and whether these methods are consistent with one another. The implementation of each technique may be dependent on the fidelity and specificity of data needed for the application, but up until now, the methods have not been compared to ensure if they can be used agonistically or not.

Aside from the capability of the technology to successfully monitor cure, there is other work to be carried out to successfully implement the technology into a production environment. For example, embedded sensors must not impact the integrity of the surrounding part [56]. One strategy is to use extremely small sensors to minimise the performance impact [84,85]. It has also been noted that a tool-mounted sensor can impact the heat transfer through a composite part depending on the tool’s material, which can potentially cause a gradient in the degree of cure [90]. Finally, there are a number of opportunities for dielectric sensors to be used for the flow monitoring of resin-infused composite parts in addition to cure monitoring. A great deal of research has been carried out to show that dielectrics can successfully capture resin arrival during an infusion process [77,78,91]. This suggests that a dielectric sensor could be used to characterise multiple process steps with a single device.

### 3.3. Ultrasonic Sensors

#### 3.3.1. Sensor Background and Governing Equations

**Figure 15:**
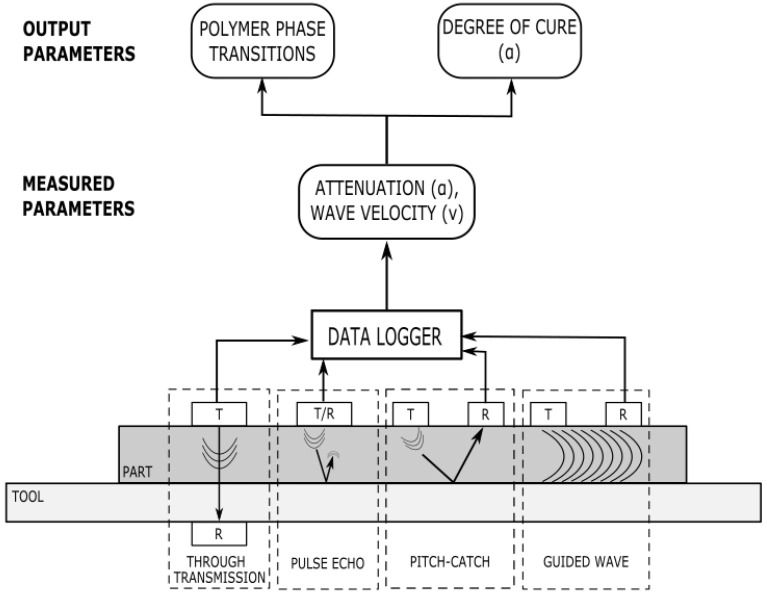
Types of ultrasonic sensors indicating how the ultrasonic waves propagate with the transmitters and receivers. The measurable parameters are linked with the cure parameters.

Ultrasonic sensor technology is commonly used for the non-destructive inspection of composite part quality and has only recently been viewed as a potential method of monitoring the cure reaction of a thermoset polymer. The main principle of ultrasonic sensor cure monitoring is that as ultrasonic waves are transmitted through the material, the propagation behaviour of these waves is impacted by the progression of the chemical reaction [92,93]. As the polymer continues to react, the density and elastic behaviour change and thus impact the velocity and attenuation of the sound waves. Multiple wave propagation models have been proposed to understand the polymer cure state [94]. All ultrasonic devices function under these principles; however, there are multiple types of transducer and receiver configurations, which are depicted in Figure 15.

The different sensor types each produce an ultrasonic wave with a measured velocity (*v*) and attenuation (*a*) characteristics, the governing equations for which are provided below. It should be noted that in literature, attenuation is commonly represented as alpha (*α*); however, here, it will be indicated by (a) to differentiate it from the definition of the degree of cure being used throughout this paper.

Longitudinal velocity (*c_L_*) is calculated in Equation (21) using the elastic modulus (*E*), velocity, and density (ρ) [33]:

(21) $$c_{L}=\sqrt{\frac{E\left(1-v\right)}{\rho \left(1+v\right)\left(1-2v\right)}}$$

The shear velocity (*c_s_*) is calculated in Equation (22) using the elastic modulus, density, and velocity [33]:

(22) $$c_{S}=\sqrt{\frac{E}{2\rho \left(1+v\right)}}$$

Attenuation is calculated in Equation (23) using the ratio of the amplitude of the incident wave (*A*) to the change of amplitude from the incident (Δ*A*) [33]:

(23) $$a=-\frac{A}{\pi \Delta A}$$

The longitudinal storage modulus (*L′)* is calculated in Equation (24) using the density, longitudinal velocity, attenuation, and wavelength (*λ*) [95]:

(24) $$\;L^{\prime }=\frac{\rho c_{L}^{2}\left(1-{\left(\frac{a\lambda }{2\pi }\right)}^{2}\right)}{{\left(1+{\left(\frac{a\lambda }{2\pi }\right)}^{2}\right)}^{2}}$$

The longitudinal bulk modulus (*L″*) is calculated in Equation (25) using density, longitudinal velocity, attenuation, and wavelength [95]:

(25) $$L^{\prime \prime }=\frac{\rho c_{L}^{2}\left(\frac{a\lambda }{2\pi }\right)}{{\left(1+{\left(\frac{a\lambda }{2\pi }\right)}^{2}\right)}^{2}}$$

The loss factor, or *tanδ*, is calculated as the ratio of the longitudinal storage and bulk moduli in Equation (26) [96]:

(26) $$\tan\delta =\frac{L^{\prime }}{L^{\prime \prime }}$$

Like dielectric cure monitoring, the parameters listed in the governing equations in this section do not correlate directly to information on material state or properties. The following section provides an overview of the correlation functions and analysis techniques that have been demonstrated in the literature to date.

#### 3.3.2. Correlation Functions

Data taken from ultrasonic sensors are commonly interpreted qualitatively, with graphic artefacts indicating polymer phase transitions that appear very similar to a DMA curve. Some varieties of ultrasonic monitoring have been referred to as ultrasonic dynamic mechanical analysis [97]. The sound waves cause molecular movement, which becomes restricted as the material becomes cross-linked. The following section is a summary of the methods that have been used in literature and includes information on the type of ultrasonic transducers and what parameters can be monitored with them.

One of the more comprehensive methods for isolating phase transitions was suggested by Lionetto et al. [97] and has been used to evaluate a polyester resin with through transmission ultrasonic monitoring. In this method, the features of the velocity versus time curve are separated into three segments:

1. Velocity is constant when the resin is liquid, but the reaction is still slow;
2. At the gel point, the velocity begins to increase, and the reaction progresses rapidly;
3. The velocity reaches a plateau at the vitrification point, indicating the slowdown of the reaction.

**Figure 16:**
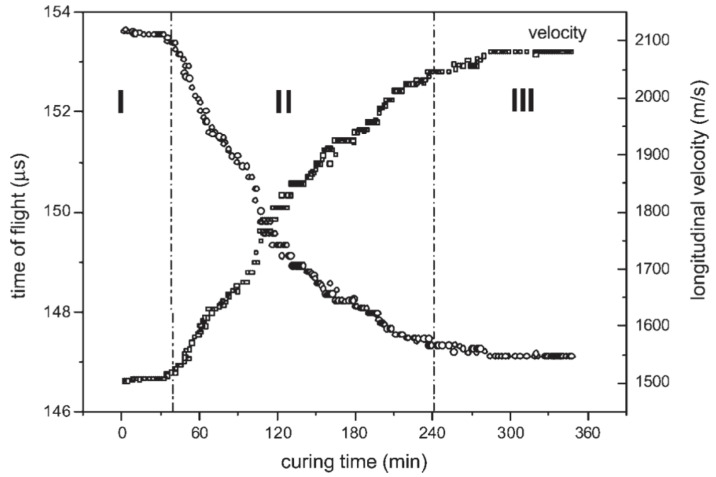
Representation of the three phases of thermoset cure based on the changes in sound velocity. Reprinted with permission from Ref. [98] 2007, John Wiley and Sons.

The distinction between these phases is shown in Figure 16, with the vertical lines indicating the approximate gel point and vitrification point.

This method of evaluating cure was also applied to the one-sided air-coupled ultrasound monitoring of polyesters [98,99] and was verified by rheological testing. This viscoelastic interpretation of phase change has also been used for the cure monitoring of epoxies using fibre-optic ultrasound sensors [100].

Ghodhbani et al. [101] used a similar method to identify the different stages of the reaction; however, this was achieved by identifying the key features of the evolution of the complex’s *c*_33_ viscoelastic coefficient throughout the curing process, with *c*_33_ being calculated by the following equation, Equation (27):

(27) $$c_{33}=\rho c_{L}^{2}(1+j\frac{2a_{L}v_{L}}{\omega })$$

in which ρ is the density, and $a_{L}$ and $v_{L}$ are the longitudinal attenuation and velocity. Once *c*_33_ can be plotted with time, the tangent method can be applied to isolate the three phases of cure:

1. The liquid viscous stage;
2. The glass transition stage;
3. The saturation solid stage.

**Figure 17:**
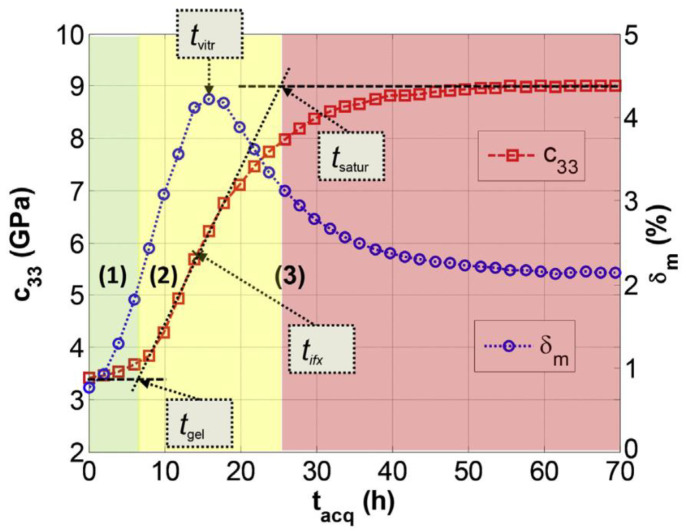
Different stages of the cure reaction based on a tangent evaluation of the complex viscoelastic coefficient, *c*_33_. Reprinted with permission from Ref. [101] 2016, Elsevier.

The transition points of *t_gel_* and *t_saturation_* are indicated in Figure 17. It should be further noted that the vitrification point can be assigned to the peak of the mechanical loss (*δ_m_*), which also roughly correlates to the inflection point of *c*_33_.

Furthermore, Ghodhbani et al. proposed a degree-of-cure model based on a Weibull distribution model of *c*_33_. The equation for the degree of cure is indicated by Equation (28):

(28) $$\alpha \left(t\right)=\frac{c_{33}\left(t\right)-c_{33,0}}{c_{33,\infty }-c_{33,0}}$$

in which the 0 and ∞ subscripts for *c*_33_ indicate the initial and maximum values. This model compared to the Kamal chemical reaction model well.

**Figure 18:**
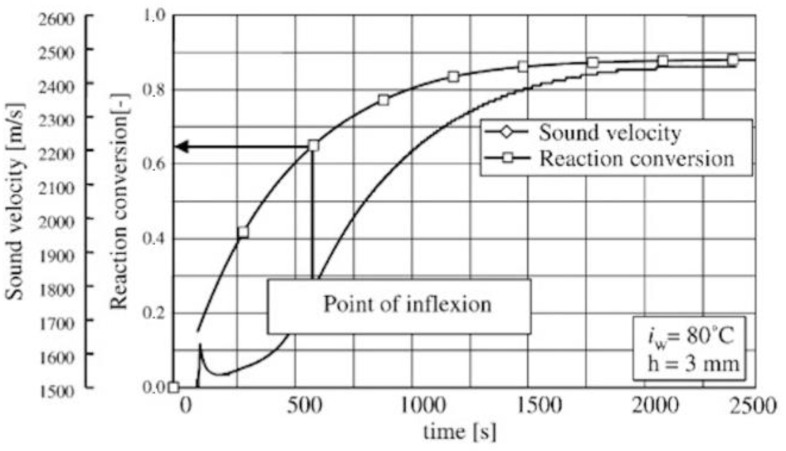
Comparison of degree of cure to the sound velocity of an epoxy-reinforced composite. Reprinted with permission from Ref. [102] 2005, Elsevier.

Schmachtenberg et al. measured the sound velocity during the infusion and cure of epoxy-reinforced fibreglass and compared it to the degree of cure calculated off-line using the DSC measurements [102]. The inflection point of the sound velocity curve was correlated to approximately 65% conversion, as shown in Figure 18.

Hudson and Yuan [103] evaluated the cure of epoxy-reinforced carbon fibres using guided-wave ultrasonic monitoring. Specifically, the group velocity of the guided waves was evaluated to determine the correlation to the cure points identified by the Convergent Raven cure simulation program.

Samet et al. [104] used attenuation to correlate to material viscosity, which was demonstrated for silicone oils. While the pulse echo configuration was not used with thermoset polymers, the equation for attenuation was shown to correlate to material viscosity, which could be used to monitor the viscosity state of a curing polymer in the future. Finding the peak attenuation has also been used to correlate to the point of vitrification for the through-transmission ultrasonic evaluation of epoxies [105] and polyesters [106].

Maffezzoli et al. [96] used a pulse echo ultrasonic transducer for the process monitoring of a thin sheet of epoxy using the longitudinal velocity and attenuation to calculate the storage and bulk moduli. The loss factor, or *tanδ*, calculated from Equation (26) was then graphed, with the peak value indicating the glass transition.

#### 3.3.3. Summary and Future Work

Ultrasonic cure monitoring may have the potential to identify cure transitions; however, this may not be sufficient for high-performance composite applications. Quality assurance requirements in the aerospace industry, for example, commonly depend on reaching a specific threshold of the degree of cure or *T_g_*, and a statement on phase transitions may be insufficient for implementation. However, ultrasonics have also been demonstrated to potentially be capable of evaluating lingering chemical reactions where dielectrics cannot [107]. In a study comparing ultrasonics, dielectrics, and nuclear magnetic resonance, the ultrasonic sensor continued to detect a response after the vitrification point of the resin where dielectric monitoring showed no activity. This could potentially indicate that ultrasonics are more sensitive, particularly in late-stage chemical reactions.

For non-destructive inspection, ultrasonics have also been demonstrated to be useful in other areas of in-process composite inspection. Scholle and Sinapius [108] demonstrated the use of ultrasonics for the cure monitoring of pultrusion processing. Multiple research groups have demonstrated that ultrasonics can successfully detect the flow front and impregnation [102,109] in addition to monitoring the thickness changes [110,111] that occur during resin infusion processing. Ultrasonics have been embedded directly into rheometric plates to collect simultaneous rheology and ultrasonic data [112]. Finally, evaluations have been conducted to capture the mechanical performance impact of embedded sensors [113]. While many ultrasonic sensors are external to the part, it is critical to understand their functional impact when they are used internally.

### 3.4. Fibre-Optic Sensors

#### 3.4.1. Sensor Background and Governing Equations

**Figure 19:**
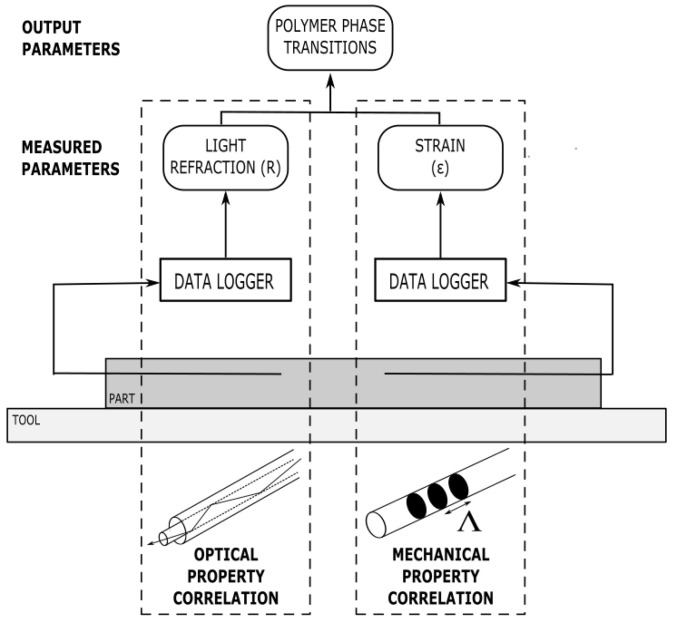
An overview of optical fibre sensing technologies, including their correlation techniques.

Fibre-optic sensors have gained attention for their use in monitoring residual strain during the thermoset cure process [89,114] and for their capabilities for structural health-monitoring in marine [115,116] and energy (wind turbine) [117] applications. The strain-monitoring capability of fibre-optic sensors has been shown to indicate phase changes during cure [81], and its potential for in situ cure monitoring has been reviewed in [117]. The two main types of optical fibres, those that detect optical properties and those that detect mechanical properties, have been reviewed in [33,34]. An overview of the types of sensing technology and their correlation techniques is show in Figure 19.

Optical fibre refractometers (OFR) utilise a cladded core fibre, in which an open portion of the core is in contact with the composite matrix material. The loss of the incident light signal is monitored based on the reflection coefficient (*R*_0_) calculated using Fresnel’s Law in Equation (29), in which *n_1_* and *n_2_*, which are the refractive indices of the core and cladding, respectively:

(29) $$R_{0}={\left(\frac{n_{1}-n_{2}}{n_{1}+n_{2}}\right)}^{2}$$

The refractive index (*n)* of the material under inspection can then be related to its density (ρ) using the Lorentz–Lorenz Law in Equation (30), in which *R_M_* is the molar refractivity, and *M* is the molar mass of the material:

(30) $$\frac{n^{2}-1}{n^{2}+1}=\frac{R_{M}}{M}\rho$$

Optical fibre interferometers (OFI), most commonly Fabry–Pérot fibres, monitor the strain imparted to the fibre by identifying the shift in the light wavelength along a series of reflective microsurfaces on the core of the fibre. The most common type of Fabry–Pérot OFI is a fibre Bragg grating (FBG) optical fibre. Under applied strain, the distance (*Λ*) between these grating changes, which then causes a shift in the Bragg wavelength (*λ_B_*). The initial Bragg wavelength is calculated via Equation (31) using the grating distance and the effective index of the fibre (*n_0_*) [81]:

(31) $$\lambda_{B}=2n_{o}\Lambda$$

The shift in the Bragg wavelength (Δ*λ_B_*) can then be calculated by Equation (32) using the initial Bragg wavelength, the strain-optic coefficient (*p_e_*), the change in strain (Δε), the coefficient of thermal expansion (*α_CTE_*), the thermo-optic coefficient (ξ), and the change in temperature (Δ*T*) [118]:

(32) $$\Delta \lambda_{B}=\lambda_{B}\left(1-p_{e}\right)\Delta \varepsilon +\lambda_{B}\left(\alpha_{CTE}+\xi \right)\Delta T$$

Equation (32) is divided into a strain-induced component of the Bragg wavelength shift and a thermal component. The decoupling of these components is an important part of interpreting the wavelength shift, as detailed in the following section, which discusses the correlation techniques for both optical property monitoring and strain monitoring.

#### 3.4.2. Correlation Functions

##### Optical Property Correlations

Fibres that monitor optical properties such as light intensity or output have been correlated to key cure events. An optical fibre with a section of cladding removed was used to monitor the cure of a bismaleimide (BMI)–carbon fibre prepreg by monitoring the attenuation of the change in light intensity [119]. In this study, the minimum attenuation was attributed to the minimum resin viscosity, the increase was attributed to the crosslinking process, and the final plateau was correlated to the end of the cure reaction, each step of which has been correlated to a numerical model.

A second study [66] used this method to evaluate the reflected light intensity of optical fibre sensors during the cure of a resin-infused carbon fibre–epoxy composite. During the infusion process, it was noted that a sharp drop in the sensor signal corresponded to resin arrival. Regarding cure, the rapid increase in the light intensity was attributed to a solidification and density increase during crosslinking, and the subsequent plateau was correlated to the end of the reaction.

A third study [120] also used this method to evaluate the refractive index of a tilted fibre Bragg grating (TFBG) optical fibre to monitor a UV-cured epoxy. In this case, an initial dip in the refractive index was attributed to the temperature response due to the exothermic reaction of the epoxy. The signal increase and plateau were then attributed to the onset of the reaction and cure completion, respectively. Similarly, an optical fibre was used to monitor the power output due to light signal changes during the cure of an epoxy resin, with the plateau of the power signal indicating the gel point [121]. The gel point was confirmed with rheology measurements.

An alternate method was used to evaluate the reflected light intensity of an FBG sensor during the cure of a graphite–epoxy prepreg [122]. In this study, the rapid increase in the reflected light intensity was also attributed to the viscosity increase due to gel and the solidification of the matrix around the fibre. However, it was noted that as the material continued to crosslink, the increase in peak intensity slows down. It was further suggested that the *T_g_* can be identified as the point where the slope of the best-fit lines for peak intensity changes. In this case, the *T_g_* determination of 95 °C agreed with the material specifications.

##### Mechanical Property Correlations

Optical fibres can also be used to monitor strain measurements using a variety of methods. The most common interpretation of the cure events follows a similar trend to the interpretation of light signals:

1. An initial dip is observed in the signal due to an increase in temperature, as the resin is still liquid and not transmitting strain to the fibre;
2. An increase in the strain measurement is observed due to the crosslinking reaction;
3. The measurement plateaus at cure completion once the matrix has frozen the fibre into place.

Multiple research groups have identified that the strain signal plateaus once the resin forms a solid matrix. An extrinsic Fabry–Pérot interferometer (EFPI) and a FBG sensor were used to identify that the strain signals level off during the vitrification phase when monitoring cure in a carbon fibre–epoxy laminate [123]. Additionally, FBG has been used to monitor a 3D braided preform infused with epoxy in which the Bragg wavelength shift was observed to plateau as the epoxy solidified [124].

**Figure 20:**
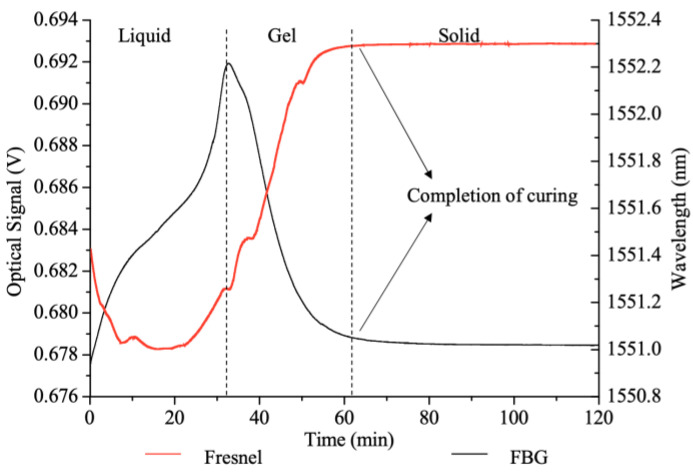
Comparison of cure behaviour for a Fresnel optical fibre sensor signal and the Bragg wavelength from an FBG signal [125].

An evaluation of epoxy cure with two varieties of optical fibres, a Fresnel optical fibre and an FBG, correlated with the results of both light and strain monitoring strategies, with a comparison of the results in Figure 20 [125]. The signal of the optical fibre was evaluated using the three-phase evaluation detailed in the previous section, whereas the Bragg wavelength identified the peak value as the onset of gel and the plateau of the signal, indicating cure completion.

A dual-period fibre Bragg grating and long-period grating (LPG) were used to monitor RTM6 epoxy cure by isolating the thermal and strain components of the Bragg wavelength shift [65]. By using two sets of gratings superimposed on the same fibre, it becomes possible to decouple the temperature and strain components. During a composite cure, there are two phases: an initial temperature ramp, at which point the resin is liquid and there is no measurable strain, and an isothermal hold, during which there is no temperature change. Using such a fibre can identify the Bragg wavelength shift as being dependent solely on the temperature component during the ramp and solely on the strain component during the dwell. Using this rationale, a 100 *μϵ* drop in strain was observed during an isothermal cure hold. The onset of this strain drop was identified as the onset of gel, and the end point of the strain drop was correlated to the end of cure. This was compared to dielectric sensor measurements collected on the same sample, which were analysed using the ion viscosity correlation, similar to the methodology used in [56] but using ion viscosity measurements.

#### 3.4.3. Summary and Future Work

Like ultrasonic sensors, at this time, fibre optics may not have the necessary quantitative output required for high-performance composite applications. While the signals can identify phase transitions in the matrix, a specific evaluation of the degree of cure is lacking. Further, it has been established that fibre-optic sensors are quite delicate and that both the embedding and the cure process have the potential to cause bending and constriction, which may negatively impact signal quality [126].

Aside from this, optical fibres show promise for residual stress measurement [122] and structural health monitoring compared to strain gauge measurement and are sensitive to changes in resin flow and mould closing during infusion processes [124]. Optical fibres can also be used to identify resin arrival and flow events during infusion processing [66,127], commonly by monitoring changes in the light signals as the length of the fibre becomes wetted by the resin [121].

**Figure 21:**
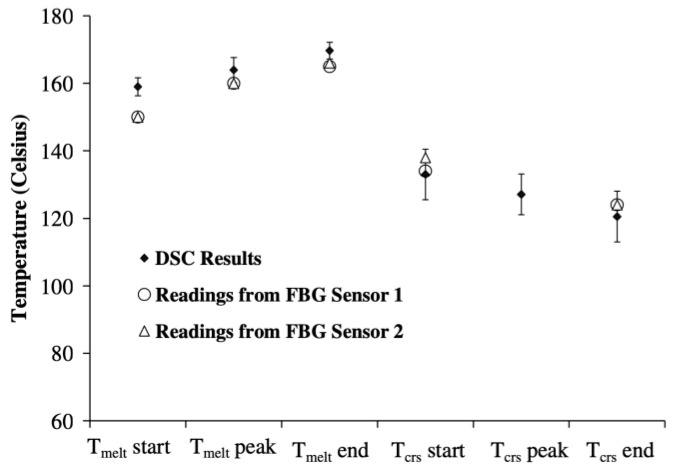
Identification of crystallisation features of a polypropylene composite using an FBG sensor compared to DSC results. Reprinted with permission from Ref. [128] 2005, Elsevier.

Finally, it is possible to monitor the crystallisation process of thermoplastic polymer by evaluating the residual strain. The processing mechanism for thermoplastic polymers is fundamentally different from the cure processing of thermosetting polymers, as they do not undergo a chemical reaction. For these materials, the sensor monitors the progression of crystallization rather than the progression of cure reaction. The Bragg wavelength shift of an FBG sensor was used to evaluate the crystallisation process for a fibreglass–polypropylene composite and successfully identified the key crystallisation points shown in Figure 21. These results were successfully compared to DSC.

## 4. Conclusions

A critical review of the correlation methods for different in-line composite cure-sensing technologies has been presented. Thermocouple cure monitoring can be reliably correlated to a degree of cure using DSC evaluation or kinetic modelling. Dielectric analysis can produce a wide variety of cure state information, as there are many correlation methods that can be applied to the different monitored parameters. Ultrasonics and fibre optics are commonly used to correlate to the specific phase transitions of the polymer rather than a quantitative measurement of cure state. While the benefits and drawbacks of implementing each type of sensor have been evaluated elsewhere, this paper asserts that it is critical to select a sensor and correlation method to achieve the required fidelity during cure monitoring for the specific application. Providing a qualitative determination of cure ending, such as fibre-optic sensors, may be appropriate for some applications. Whereas an application which requires a degree of cure or *T_g_* with a specific value may benefit from thermocouple or dielectric sensing.

There are multiple areas of potential improvement for in situ cure-sensing technology. The availability of non-invasive sensors and sensors that do not require a permanent installation would increase the ease of implementation. The development of a quantitative measure of cure for sensors, such as ultrasonics, would enable their use in a wider range of applications. A comparison of the different correlation methods for each sensor type would identify the most accurate method for evaluating cure progress, including whether the methods are applicable across multiple materials and multiple cure cycles. Finally, a robust evaluation of the correlation methods across repeated process cycles would indicate if the precision was sufficient to capture manufacturing variations. Future work in these areas would improve the fidelity of data collection and enable new sensing technologies to be readily and confidently adopted.

## Data Availability

The data presented in this study are available on request from the corresponding author.
